# 2024 Acknowledgment of *mSystems Ad Hoc* Reviewers

**DOI:** 10.1128/msystems.01628-24

**Published:** 2025-01-21

**Authors:** Jack A. Gilbert

**Affiliations:** 1 University of California San Diego School of Medicine, La Jolla, California, USA

## EDITORIAL

As we reflect on the challenges and triumphs of this year, it is clear that the global landscape—both political and scientific—is more complex than ever. In an era marked by rapid technological advancements, political uncertainties, and the evolving dynamics of societal trust in science, the role of rigorous peer review has never been more vital. Scientific integrity is not just an academic ideal; it is a foundation upon which informed decisions are made, lives are saved, and futures are shaped. Peer review stands as a cornerstone of this integrity, ensuring that each piece of published research withstands the scrutiny of experts and adheres to the highest standards of accuracy and reliability. Yet, the system itself depends on the dedication of reviewers who balance this critical responsibility with their own research, teaching, and personal lives. Their contributions are not merely procedural—they are acts of resilience and commitment that fortify the trustworthiness of science in a time when such trust is paramount. This year, as *mSystems* continues to grow as a hub for groundbreaking microbiome and systems biology research, we are reminded that the act of peer review transcends the pages of our journal. It is a testament to the collective determination of a global scientific community to rise above uncertainty and uphold the principles of collaboration, mentorship, and truth. In the face of mounting pressures—be it from overburdened workloads, geopolitical instability, or societal skepticism—your efforts as reviewers ensure that science remains a beacon of clarity and progress. To every reviewer who has contributed their time, expertise, and thoughtful critique, we offer our deepest gratitude. You are not just gatekeepers of rigor; you are architects of a future where science continues to be a trusted guide for humanity. Your dedication to this process exemplifies the best of what we, as a community, can achieve. In this, my last year as EIC of *mSystems*, and my last chance to extoll the virtues of peer review, I want to thank you all personally for your invaluable service to *mSystems,* to the American Society for Microbiology, and to the greater scientific ecosystem. Together, we navigate the complexities of our world, ensuring that the legacy of scientific excellence remains unwavering for generations to come.

On behalf of the editors of *mSystems*, I gratefully acknowledge the following individuals, who served as reviewers for the journal from November 2023 through November 2024. Their time and effort in reviewing articles have been essential in ensuring the high quality of our publications, and their help is greatly appreciated.

Aula Abbara

Ghazanfar Abbas

Sophie Saphia Abby

Rehab Z. Abdallah

Masahiro Abe

Tjakko Abee

Florence Abravanel

Sabrina Absalon

Tomaz Accetto

Barbara I. Adaikpoh

Walter Adams

Kamoru A. Adedokun

Innocent Afeke

Ruth Aguila-Ramírez

Emily G. Aguirre

Md Ahaduzzaman

Tae-Hyuk Ahn

Jewelna Akorli

Md Tauqeer Alam

Luis D. Alcaraz

Alison Felipe Alencar Chaves

Gladys Alexandre

Alexandra Alexiev

Madeline Alizadeh

Adel Al-Janabi

Celeste Allaband

Alexander B. Alleman

Kylie Allen

Salvador Almagro-Moreno

Qadreyah A. Almatawah

Rashid Alrahbi

Sudheer Kumar Aluru

John Alverdy

Samat Amat

Heather K. Amato

Israel Dave Ambita

Tessa Andermann

Alexander C. Anderson

Matthew Zack Anderson

Sean Anderson

Haike Antelmann

Hiroshi Arai

Stephane Aris-Brosou

Holly K. Arnold

Janelle C. Arthur

Mani Arumugam

Abigail Esenam Asangba

Aman Ashar

Melinda Marie Ashcroft

Philip M. Ashton

Francesco Asnicar

Joseph Atia Ayariga

Frank O. Aylward

Rajeev Azad

Hamed Azarbad

Taj Azarian

M. Andrea Azcárate-Peril

Sheyda Azimi

Houda Baati

Heidi Bai

John F. Baines

Djordje Bajic

Frederik Bak

Izzy Baker

Sophia Bałdysz

Gregory Ballash

Sreejata Bandopadhyay

Aparna Banerjee

Fernando Baquero

Daniel Barkan

John Barlow

Francisco Barona Gómez

Lars Barquist

Didier Barradas-Bautista

Arianna Basile

Tiffany Batarseh

Anthony D. Baughn

Carla Bautista Rodriguez

Clifford J. Beall

William N. Beavers

Gregg T. Beckham

Payam Behzadi

Peter Belenky

Aeriel D. Belk

Corinne Benakis

Mahmoud Bendary

Jose A. Bengoechea

Alfonso Benítez-Páez

Mikkel Bentzon-Tilia

Hanna Leah Berman

Patricia Bernal

Eryn E. Bernardy

Louis Berrios

Stefan Bertilsson

Matthew Bertin

Maria T. Bertoli

Yunus Emre Beyhan

Punyasloke Bhadury

Tamanash Bhattacharya

Sunanda Bhattacharyya

Gabriele Bianco

Haijian Bing

Xiaoli Bing

Karishma Bisht

Pradeep Bist

Indranil Biswas

Aaron Bivins

Alfredo Blakeley-Ruiz

Alissa Bleem

Matthew Blow

Maksym Bobrovskyy

James Boedicker

Jamie Bojko

Zsolt Boldogkői

Elisa Borghi

Karsten Borgwardt

Yann F. Boucher

Paul Boudreau

Salim Bougouffa

Patric Bourceau

Patrick H. Bradley

Christian Braendle

Jason M. Brenchley

Colin J. Brislawn

Ana Rita Brochado

Julie A. Brothwell

Michael S. M. Brouwer

Bryan Paul Brown

James R. Brown

Jan David Brüwer

Mark P. Brynildsen

Carmen Buchrieser

Shelly Buffington

Michal Bukowski

Davide Bulgarelli

Kristin M. Burkholder

Jonathan T. Busada

Mariaelena Caboni

Elisabetta Cacace

Lindsay K. Caesar

Tânia Caetano

Jingyi Cai

Zhendong Cai

Benjamin Calfee

Carole Camarasa

Andrew Cameron

Andrew D. S. Cameron

Gerard A. Cangelosi

Anahi Cantoran

Wenzhi Cao

Huizhen Cao

Mauricio Caraballo Rodriguez

Michael Carlson

Ross P. Carlson

Tyler J. Carrier

Monica Cartelle Gestal

Kayla A. Carter

Michele Castelli

Ezequias Castillo-Lopez

Santiago Castillo-Ramírez

Jorge Cervantes

Miguel Angel Cevallos

Anirban Chakraborty

Douglas Chalker

Mathias Chamaillard

Julien Chamberland

Matthew Champion

Patricia A. Champion

Lena Champlin

Jason Chan

Jung Chang Hwa

Trevor C. Charles

Susanta Chatterjee

Santanu Chattopadhyay

Koel Chaudhury

Tanya Cheeke

Biao Chen

Ce Chen

Congying Chen

Dong Chen

Jie Chen

Jun Chen

Lei Chen

Liang Chen

Lianmin Chen

Linxing Chen

Muxuan Chen

Peigen Chen

Qu Chen

Ruixi Chen

Stanley Chen

Xiangliang Chen

Ya-Jou Chen

Yan Chen

Yanfei Chen

Yihua Chen

Zhen Chen

Zhi Chen

Lei Cheng

Qiwen Cheng

Yin-Ru Chiang

Byung-Kwan Cho

You-Hee Cho

Lon M. Chubiz

Le Van Chuong

Claudia Claus

Alison Coady

José Francisco Cobo Díaz

Maureen L. Coleman

John J. Colgan

Andre M. Comeau

Jacqui Comstock

Sarah S. Comstock

Bruno Contreras-Moreira

Perran Cook

Ryan Cook

Kevin M. Coombs

Florencia Correa-Fiz

Richard Costa Polveiro

Pauline M. L. Coulon

Axel Cournac

Chris Creevy

Alex Crits-Christoph

Bo Cui

Jie Cui

Matthew J. Culyba

Scott Cunningham

Tom Curtis

Ankur B. Dalia

Doralyn S. Dalisay

Marion Dalmasso

F. Darfeuille

Sudip Das

Lawrence A. David

Maude David

Michael Z. David

Jean Debedat

Katherine Deets

Ronnie De Jonge

Tom Delmont

Ilenne Del Valle

Fei Deng

Patricia Diaz

Katja Dierking

Nicholas Dimonaco

Sara Di Rienzi

Shaillay Dogra

Terje Dokland

Stephen K. Dolan

Claudio Donati

Nadezhda Tsankova Doncheva

Hao Dong

Jiu-Hong Dong

Annie Donoghue

Matthew Doremus

Marisol I. Dothard

Daolong Dou

Jordan Dowell

Andrew C. Doxey

Charles M. Dozois

Glen D'Souza

Hong Du

Zhibing Duan

Bhumika Dubay

Leonard Dubois

James Gabe Dubose

Keith Dufault-Thompson

Parambir Dulai

Sylvia Duncan

Anne K. Dunn

Micah Dunthorn

Rachel J. Dutton

Jonathan Dworkin

Emily Ebel

Hanareia Ehau-Taumaunu

John Eid

Sahar El Aidy

Joshua E. Elias

Melissa Ellermann

Mostafa S. Elshahed

Brandon C. Enalls

Mark C. Enright

Hyungjin Eoh

Aaron Conrad Ericsson

Andrew James Fabich

Camilla Fagorzi

Chia-Kwung Fan

Huizhou Fan

Linchuan Fang

Na Fei

Limin Feng

Xiaowen Feng

Lorena Fernandez

Valentina Ferrari

Edgar Francisco Ferrer-Gonzalez

Giulio Ferrero

Pamela Ferretti

Ilario Ferrocino

Jose Ferronatto

David B. Fidler

Daniel Figeys

Melanie J. Filiatrault

Cara Fiore

Christopher D. Fjell

Avi I. Flamholz

Citlali Fonseca Garcia

Herrison Fontana

Ebenezer Forster-Nyarko

Leonard J. Foster

Eric A. Franzosa

Elliot S. Friedman

Thilo M. Fuchs

Susana Fuentes

Jed Fuhrman

Gavin Fujimori

Heather Fullerton

Maximilian Furst

Tara N. Gaire

Fang Gan

Erika Ganda

Michael G. Ganzle

Feng Gao

Guangliang Gao

Yuan Gao

Brianna Garcia

Erin C. Garcia

Sarahi L. Garcia

Diego Garcia De La Morena

José Manuel García-Fernández

Hernan Garcia-Ruiz

Nisha J. Garg

Anna Garona Delgado

Daniel Rios Garza

Benedikt Geier

Emily Gentry

Robin Gerlach

Travis Gibson

Abraham Gihawi

Rosario Gil

Steven R. Gill

Sandra Gillespie

Nils Giordano

Giorgio Giraffa

Stefano Giulieri

Emile Gluck-Thaler

Filipa Godoy-Vitorino

Casey Godwin

Yanhai Gong

Fernando Gonzalez-Candelas

Graciela Gonzalez-Gil

Ana Camila Gonzalez-Nayeck

Benjamin Good

Frédéric Goormaghtigh

Isabel Gordo

Susan Gottesman

Friedrich Götz

Richard L. Gourse

Alexandre Gouzy

Grzegorz Jan Grabe

Sarah M. Gray

Leigh Greathouse

Lucia Grenga

Pranas Grigaitis

Antonio Grimalt-Alemany

Ghjuvan M. Grimaud

Teddy Groves

Katarzyna Grzelak-Błaszczyk

Bing Gu

Unnur Gudnadottir

Alejandro Guerrero-Lopez

Ghita Guessous

Karen Guillemin

Nesibe Selma Güler Çetin

Dipika Gupta

Serafin Gutierrez

Tamar Guy-Haim

Hyun Soon Gweon

Elaine M. Haase

Abderrahman Hachani

Timothy J. Hackmann

Anna Hakobyan

Ruth M. Hall

Tobin Hammer

Mei-Ling Han

Shuo Han

Xiao Han

Mohammed Hankir

Shuai Hao

Miyuki Harada

Elizabeth L. Harvey

Nur A. Hasan

Fumihito Hasebe

Kristina Haslinger

Karl A. Hassan

Heidi Hau

Jane Hawkey

Gang He

Tao He

Zhibin He

Marco Hein

Almut Heinken

Eric Jan Nikolaus Helfrich

Antoni P. A. Hendrickx

Tory A. Hendry

Matthew Henke

Christopher Henry

Michael Henson

Jennifer Heppert

Marta Hernández-García

Obed Hernandez-Gomez

Robert L. Hettich

Krzysztof Hinc

Ying-Ning Ho

Joanne K. Hobbs

Andrew J. Hoisington

Matthew T. G. Holden

Devin B. Holman

Johanna Holman

Yoichi Honda

Suzie Hoops

Cristina Howard-Varona

Daniela Hozbor

Ansel Hsiao

Fupin Hu

Jiankun Hu

Shuai Hu

Yichen Hu

Peng Huang

Qiang Huang

Shucheng Huang

Weijun Huang

Ziyuan Huang

Gary B. Huffnagle

Dana E. Hunt

Neil Hunter

Myung Hwangbo

Alexander N. Ignatov

Hiroyuki Imachi

Muhammad Imran

Ikbal Agah Ince

Zahra Fatima Islam

Dawn A. Israel

Dandan Izabel-Shen

Jonathan L. Jacobs

Jeonghwan Jang

Elisabeth M.-L. Janssen

Laura R. Jarboe

Scott A. Jarmusch

Benjamin Anderschou Holbech Jensen

Paul A. Jensen

Baolei Jia

Chao Jiang

Haibo Jiang

Hongchen Jiang

Xiaofang Jiang

Yu Jiang

Shuo Jiao

Hao Jin

Meng Jin

Yu Jin

David R. Johnson

Philip L. F. Johnson

Timothy A. Johnson

Bradley D. Jones

Eric Jones

Jeremy C. Jones

Korin Rex Jones

Marie Joossens

Quentin Josso

Luke R. Joyce

Feng Ju

Lisa Kahl

Olga V. Kalinina

Alice Kane

Hyun Ah Kang

Nagarajan Kannan

Rose S. Kantor

Anna Karnkowska

Lisa Karstens

Kazuyuki Kasahara

Eisuke Kato

Yisehak Kechero

Thomas E. Kehl-Fie

Scott T. Kelley

Cheryl A. Kerfeld

Eman M. Khalaf

Mohammadali Khan Mirzaei

Olga E. Khokhlova

Hyeun Bum Kim

Jaebeom Kim

Joonhoon Kim

Michiel Kleerebezem

Manuel Kleiner

Anke Klueter

Uli Klümper

Gwenan M. Knight

Travis Kochan

Travis Joseph Kochan

Coco Koedooder

Benjamin J. Koestler

Ryuichi Koga

Fotini Kokou

Glynis L. Kolling

Ilana Kolodkin-Gal

Aleksandra Kołodziejczyk

Weidong Kong

Konstantinos T. Konstantinidis

Tal Korem

Tomasz Kosciolek

Erika Kothe

Ariangela J. Kozik

Cristina Kraemer Zimpel

Karin E. Kram

Gabriela Bergiante Kraychete

Marie Kroeger

Ines Krohn

Sergey Kryazhimskiy

Karl Kuchler

Ursula Kües

Anand Kumar

Rolf Kümmerli

Benoit Kunath

Barbara N. Kunkel

Julia Maria Kurth

Denis Kutnjak

Jessica M. Labonte

Kurt Labutti

Leo Lahti

Henry Lam

Richard J. Lamont

Lefu Lan

Ping Lan

Cristina Landeta

Guillaume Larivière-Gauthier

Alyse Larkin

Ewa Laskowska

Jason Laurich

Amit Lavon

Janice E. Lawrence

Christopher E. Lawson

Simon Lax

Shuai Le

Daniel R. Leadbeater

I. J. Lean

Andrea Lear

Sam Leareng

Francois Lebreton

Quentin J. Leclerc

Charles Kai-Wu Lee

Vincent T. Lee

Sonja Lehtinen

Kyle R. Leistikow

Ylva Lekberg

Jose M. Lemme Dumit

Jay T. Lennon

Lex E. X. Leong

Pok Man Leung

Petra Anne Levin

Erel Levine

Asaf Levy

Kim Lewis

Michael Lewis

Ting Li

Guanhong Li

Juan Li

Junhui Li

Le Li

Robert W. Li

Ruichao Li

Xi Li

Xian-Zhi Li

Yanhua Li

Yong Li

Yongxin Li

Zhe Li

Zhipeng Li

Zhuang Li

Tian Liang

Chen Liao

Ningbo Liao

Xiudong Liao

Stephen J. Libby

Tami Lieberman

Ciriaco Ligios

Shuangjun Lin

Nilton Lincopan

Eva Lindsröm

Zhang Li-Qun

Bin Liu

Dejun Liu

Fengping Liu

Haoran Liu

Jinxin Liu

Jiwen Liu

Lei Liu

Liyun Liu

Qin Liu

Shuting Liu

Yong-Xin Liu

Yongqin Liu

Yongxin Liu

Yuanyuan Liu

Kai Liu

Krista Longnecker

Gamaliel López-Leal

Alexander Loy

Gabriel L. Lozano

Catherine Lozupone

Huijie Lu

Junfeng Lu

Kuan-Yi Lu

Tao Lu

Yang Lu

Fan Lü

Jose Lugo-Martinez

Sara Lundgren

Xuesong Luo

Mor N. Lurie-Weinberger

Bin Ma

Teng Ma

Boris Macek

Daniel Machado

Enea Maffei

Ashish Malik

Sairah Malkin

Joanna Malukiewicz

Nathan P. Manes

Sam Manna

Prasanth Manohar

Thomas Joseph Mansell

Yongjiang Mao

Esteban Marcellin

Marco Mariotti

Margaret Mars Brisbin

Matthias Marti

Jose Manuel Martí

Ana B. Martin-Cuadrado

Joval Navas Martinez

Tamara Martin-Pozas

Mathias Martins

Samuel J. Martins

Joleen Masschelein

Ruth Massey

William Joseph Massey

Jana C. Massing

Molly A. Matty

Mauro Maver

Tim McAllister

Daniel McDonald

Michael McDonald

Simon McIlroy

Katherine McMahon

Sean Meaden

Conor Meehan

Joshua Chang Mell

Yixuan Meng

Guillaume Méric

Matthias Merker

Laura A. Mike

Jeremiah J. Minich

Kazumori Mise

Kate Miska

Ljubisa Miskovic

Sara Mitri

Jennifer M. Mobberley

Samuel J. Modlin

Katarzyna Modrzynska

Andrew H. Moeller

Hosein Mohimani

Wendy W. K. Mok

Nima Montazeri-Najafabady

Peter Kotsoana Montso

Soumya Moonjely

Andy Moorehead

Zayda Morales Moreira

Cristina Moraru

Ana Paula B. Moreira

Andrey Morgun

Taiki Mori

Lucas Morinière

Paul Morley

Robert M. Morris

Erick V. S. Motta

Jean-Paul Motta

Walaa K. Mousa

Izabela Mujakic

Juan Francisco Mujica-Alarcon

Cécile Muller

Rolf Müller

Marcelo Müller-Santos

Conrad W. Mullineaux

Mikayla Murphree-Terry

Mario E. Muscarella

Dorota Muth-Pawlak

Jillian Myers

Indira U. Mysorekar

Alessio Naccarati

Kentaro Nagaoka

László Nagy

Sue C. Nang

Takashi Narihiro

Jalees A. Nasir

William Wiley Navarre

Laura Emilce Navas

K. H. M. Nazmul Hussain Nazir

Jacob T. Nearing

David E. Nelson

Mihai Netea

Irene L. G. Newton

Sherrianne Ng

Nguyen T. Q. Nhu

Kuikui Ni

Yan Ni

Mark Nicol

Jeppe Lund Nielsen

Mikael Niku

Ben Niu

Deng-Ke Niu

Justin R. Nodwell

Cecilia Noecker

Juan Nogales

Niclas Nordholt

Jeanette M. Norton

Noelle Noyes

Erin Nuccio

Takuro Nunoura

Nathan Nuzum

Toshihiro Obata

Howard Ochman

Erkison Ewomazino Odih

Kieran O'Doherty

Kazuhiro Ogai

Marco Rinaldo Oggioni

Amanda G. Oglesby

Chang-Sik Oh

Robin A. Ohm

Barry O'Keefe

Ole Andreas Økstad

Stephen G. Oliver

Alejandro Olmedo-Velarde

Kimiho Omae

Takashi Ono

Ivan Oresnik

Carlos J. Orihuela

Mehmet A. Orman

William D. Orsi

Megan Orzalli

Ebrahim Osdaghi

Birge D. Özel-Duygan

Baozhu Pan

Xuming Pan

Zhiyuan Pan

Ivona Pandrea

Kevin Panke-Buisse

Suresh Panthee

Jason A. Papin

Ben Pascoe

Alessandro Passera

Sweta M. Patel

Talima Pearson

Nicole Danielle Pecora

Shyamal Peddada

Luis Pedro Coehlo

Rafael Pena-Miller

Bo Peng

F. Christopher Peritore-Galve

Natalia Borisovna Perunova

Rita de Cassia Pessotti

William A. Petri

Veronika K. Pettersen

Jennifer Philips

Bernadeta Pietrzak

Erica Louise Plummer

Claudia Pogoreutz

Katherine S. Pollard

Shawn W. Polson

Ines Pons

Ana Pontes

Phillip G. Popovich

Lance B. Price

Alexander J. Probst

Paulina Prondzinsky

Tanel Punga

John G. Purdy

Fahd Qadir

Xiaozhou Qi

Yaping Qi

Guoliang Qian

Luke Qian

Xinyun Qiu

Lynne M. Quarmby

Zachary Quinlan

Christopher D. Radka

Sanguthevar Rajasekaran

Juan David Ramirez

Marcel Ramirez

Kathryn M. Ramsey

Tara M. Randis

Xiancai Rao

Thomas Rattei

Isabella Rauch

Chinnarajan Ravindran

J. Christian J. Ray

Timothy D. Read

F. Jerry Reen

Mujeeb Ur Rehman

Alexandra Rehn

Olaya Rendueles-Garcia

Hamid Reza Mollaei

Francesco Ricci

Simon K.-M. R. Rittmann

Damian W. Rivett

Edson Rocha

Katherine Roche

Luis Roderiguez-R

Andjela Rodic

Eduardo Rodríguez-Román

Louise Roer

TJ Rogers

Fernando Rojo

Antonis Rokas

Benjamin R. K. Roller

Pascale Romby

Jesus A. Romo

Stephanie M. Rosales

David A. Rosen

Benjamin Ross

Corinna Ross

Omar Rota-Stabelli

Alissa Rothchild

Simon Roux

Sarah Elizabeth Rowe

Denis Roy

Piklu Roy Chowdhury

Taniya Roy Chowdhury

Thomas Rudel

Antoinette Russell

Ben Sadd

Sabrina Sadiq

Rajib Saha

Sandhini Saha

Yasubumi Sakakibara

Ali Salehzadeh-Yazdi

Samantha Saleri

Steven L. Salzberg

Kris Sankaran

Alvaro San-Milán

Tasha Marie Santiago-Rodriguez

Janko Sattler

Matthew Scarborough

Peter J. Schaap

Herb E. Schellhorn

Sergio Schenkman

Patrick Schimmel

Patrick M. Schlievert

Patrick D. Schloss

Stephan Schmitz-Esser

Sijmen Schoustra

Gerrit J. Schut

Hannah Doris Schweitzer

Alison J. Scott

Bruce Seal

Craig See

Rebecca Segal

Michael Seidl

Jana Seifert

William Self

Mohammad R. Seyedsayamdost

Justin Shaffer

Stephanie R. Shames

B. Jesse Shapiro

Thomas J. Sharpton

Xihui Shen

Hengbo Shi

Jianxin Shi

Pixu Shi

Dylan Shropshire

Cheryll Sia

Luciana Damascena Silva

Justin D. Silverman

Yogendra Singh

Federico Sisti

David Skurnik

Robert P. Smith

Stephanie N. Smith

Kim Sneppen

Scott D. Soby

Michael B. Sohn

Christophe Sola

Vincent Somerville

Bongkeun Song

Chunxu Song

He Song

Hyun-Seob Song

Vijay Soni

Utkarsh Sood

Stijn Spaepen

Sean Spencer

Rachel L. Spietz

Robin Spiller

Sushmita Sridhar

Kim Stanford

Ken Stedman

Martin Steinegger

Lukasz L. Stelinski

David Cole Stevens

Bram W. G. Stone

Mikael Lenz Strube

Reed M. Stubbendieck

Xiaoquan Su

Yong Su

Jordy Evan Sulaiman

Jian Sun

Jun Sun

Shan Sun

Xingmin Sun

Yanni Sun

Yukun Sun

Zhihong Sun

Way Sung

Susheel Susheel

Sanjay Swarup

Stephen J. Taerum

Prafullakumar Tailor

Akinori Takaoka

Tamara Tal

James Tabi Tambong

Jie Tan

Biao Tang

Hongzhi Tang

Julien Tap

Benno H. Ter Kuile

Malak Tfaily

T. Frede Thingstad

Sondra C. Thurjeman

Liang Tian

Raul Tito

Andrzej Tkacz

Kelvin Kai-Wang To

Bradley B. Tolar

Dongdong Tong

Joshua Tran

Anke Trautwein-Schult

Todd Treangen

Binu Tripathi

Gareth Trubl

Tanya Tschirhart

Boo Shan Tseng

Pablo Tsukayama

Hein-Min Tun

Lucas J. Ustick

Gupta Vadakattu

Agustin Valenzuela-Fernández

Miguel A. Valvano

Peter van Baarlen

Wannes Van Beeck

Anne van der Meij

Adrianus W. M. van der Velden

Sue Vandewoude

Marc Van Goethem

Lynn Vanhaecke

Stineke van Houte

James T. Van Leuven

Laurence Van Melderen

Willie F. Vann

Máté Vass

Nic M. Vega

Ben Vezina

Evgeny Vinogradov

Max von Kleist

Jay Vornhagen

Irene Wagner-Dobler

Catherine Ann Wakeman

Seth T. Walk

Alan W. Walker

Kimberly A. Walker

Aaron M. Walsh

Calum Walsh

David A. Walsh

Baikui Wang

Baohong Wang

Bo Wang

Chengshu Wang

Gang Wang

Hongmei Wang

Hsin-Yao Wang

Jiawei Wang

Jichao Wang

Jingjie Wang

Junjun Wang

Shuai Wang

Xiang Wang

Xinxia Wang

Yanan Wang

Ying Wang

Yongzhong Wang

Yuehong Wang

Akaraphol Watcharawipas

Paula I. Watnick

Cassandra J. Wattenburger

J. Scott Weese

Yuqiu Wei

Alaina Weinheimer

Alexandra J. Weisberg

Philipp Wendering

Guido Werner

Ruud A. Weusthuis

William B. Whitman

Emma Whittle

Ryan R. Wick

Kara Wiggin

Kiara Wiggins

H. Steven Wiley

Ian Will

Candace L. Williams

Lukas Wisgrill

Christopher Witzany

Benedykt Wladyka

Felix Wong

A. Jamie Wood

Jing Wu

Shengru Wu

Yi Wu

Xi Xia

Yu Xia

Xiao Xiao

Fei Xie

Yuwei Xie

Peng Xu

Qingbiao Xu

Xiaoling Xu

Xin Xu

Zhenjiang Zech Xu

Chen Xue

Hone Xue

Shuhei Yabe

Srujana Samhita S. Yadavalli

Abbas Yadegar

Jiangwei Yan

Jiuliang Yan

Ming Yan

Jian Yang

Julianne Yang

Qian Yang

Ruifu Yang

Yaohua Yang

Mingfei Yao

Yu-Feng Yao

Min Yap

Wee Wei Yee

Zhao Yicheng

Wen-Bing Yin

Sung Ho Yoon

Lingchong You

Mingdan You

Vincent Young

Fangyou Yu

Hang Yu

Xiaofei Yu

Yunsong Yu

Yunsong Yu

Alex Yuan

Jun Yuan

Joseph P. Zackular

Naufal Zagidullin

Merve S. Zeden

Takeshi Zendo

Lanying Zeng

Xian Zeng

Yue Zeng

Yong Everett Zhang

Angela Zhang

Chengcheng Zhang

Dao-Feng Zhang

Faming Zhang

Heping Zhang

Hua Zhang

Jie Zhang

Lingdi Zhang

Meng Zhang

Ran Zhang

Yanying Zhang

Yongjun Zhang

Zhigang Zhang

Haixiang Zhang

Guang Zhao

Kun Zhao

Zhe Zhao

Olga Zhaxybayeva

Hao Zheng

Meiqin Zheng

Qingfei Zheng

Weibin Zheng

Xiaowei Zheng

Xuhui Zheng

Kevin Xu Zhong

Dongsheng Zhou

Ning-Yi Zhou

Xuguo Zhou

Yingshun Zhou

Zhemin Zhou

Zunchun Zhou

Bofeng Zhu

Yan Zhu

Jackie Zorz

Juanjuan Zou

Ling Zou

Simone Zuffa

Tao Zuo

